# Entomopathogenicity and Biological Attributes of Himalayan Treasured Fungus *Ophiocordyceps sinensis* (Yarsagumba)

**DOI:** 10.3390/jof3010004

**Published:** 2017-02-05

**Authors:** Bikash Baral

**Affiliations:** 1 Research, Community Development and Conservation Center (C3DR), Pokhara 33700, Nepal; bikash.baral@utu.fi or bikubaral@yahoo.com; Tel.: +358-468-415-353; 2 Department of Biochemistry, University of Turku, Turku, Finn-20014, Finland

**Keywords:** Fungal elicitor, MAP kinase, metabolites, PR-proteins, receptors, signal transduction, transcriptional factors, transcriptome sequencing, virulence factors

## Abstract

Members of the entomophagous fungi are considered very crucial in the fungal domain relative to their natural phenomenon and economic perspectives; however, inadequate knowledge of their mechanisms of interaction keeps them lagging behind in parallel studies of fungi associated with agro-ecology, forest pathology and medical biology. *Ophiocordyceps sinensis* (syn. *Cordyceps sinensis*), an intricate fungus-caterpillar complex after it parasitizes the larva of the moth, is a highly prized medicinal fungus known widely for ages due to its peculiar biochemical assets. Recent technological innovations have significantly contributed a great deal to profiling the variable clinical importance of this fungus and other related fungi with similar medicinal potential. However, a detailed mechanism behind fungal pathogenicity and fungal-insect interactions seems rather ambiguous and is poorly justified, demanding special attention. The goal of the present review is to divulge an update on the published data and provides promising insights on different biological events that have remained underemphasized in previous reviews on fungal biology with relation to life-history trade-offs, host specialization and selection pressures. The infection of larvae by a fungus is not a unique event in *Cordyceps*; hence, other fungal species are also reviewed for effective comparison. Conceivably, the rationale and approaches behind the inheritance of pharmacological abilities acquired and stored within the insect framework at a time when they are completely hijacked and consumed by fungal parasites, and the molecular mechanisms involved therein, are clearly documented.

## 1. Introduction

The caterpillar-parasitizing fungus *Ophiocordyceps sinensis* (Berk.) G.H. Sung, J.M. Sung, Hywel-Jones & Spatafora (syn. *Cordyceps sinensis* (Berk.) Sacc.) is an insect-borne fungus, widely known as “Chinese caterpillar fungus” in English [1] and “Yarsagumba (fungus *cum* larvae)” in Nepali. Taxonomic classification places the organism within the phylum Ascomycota (sac fungi), the family Ophiocordycipitaceae in the order Hypocreales [2,3,4], and it is often referred to as an entomophagous fungus owing to its parasitic nature in insects’ larvae [4,5]. The fungus possesses wide host range, killing Lepidopteran larvae of more than 60 different species [6]. Although it can infect 30 of 40 known species of Thitarodes caterpillars [7], the Himalayan bat moth *Hepialus armoricanus* proves to be a usual and common host for this fungus [6]. Entomopathogens such as *Beauveria bassiana* and *Metarhizium robertsii* which belong to Hypocreales of Ascomycota are known to be the best-studied models to decipher the exact mechanism of the biological and physiological interactions, and thus these fungi are also widely used as biopesticides [8]. Unlike the broad-host-spectrum entomopathogen *M. robertsii*, other fungi such as *M. acridum* and *M. majus* display more specificity, respectively, for locusts and beetles [9].

## 2. *Cordyceps* Ecology

Fungi such as *O. sinensis* (abbreviated as *OS* in the subsequent text) are concentrated at a very high altitude (>4000 m above sea level) [10], with a capacity to endure the frigid climate of the high Nepalese Himalayas and the Tibetan Plateau [1,11,12]. Owing to restricted localization in the higher altitudes (alpines) and Tibetan areas, it has now been presumed as a flagship fungus of China [13]. Wide-spread coverage of this fungus (so-called Himalayan gold) also occurs in different high-altitudinal Nepalese Himalayas, from where a large chunk of *Cordyceps* are collected and exported every year, however, mainly through the illegal routes [14]. Wide distribution of *Cordyceps* sp. occurs in 27 out of 75 districts of Nepal, while the Darchula and Dolpa districts are the most prominent ones (in terms of distribution). Ideal pharmacological capabilities and higher economic returns immediately after its harvest, however, have caused tremendous over-exploitation, enlisting it as a threatened species in the red book (for detail economics review, see [14]).

Among over 1200 different animal and insect pathogenic fungi known [15], *Cordyceps* stands up as the largest genus in the entomopathogens list, comprising almost 500 species and varieties [16,17,18]. Beside the fact that entomopathogens have evolved an amazing and remarkable diversity of infection modes and nutritional strategies, the underlying signaling cascades encoding fungal pathogenicity factors seem to be well conserved within different *Cordyceps* species. The Lepidopteran larvae prove to be the preferred hosts for *Cordyceps* infection, while insects belonging to Coleoptera, Orthoptera, Hemiptera and Hymenoptera orders are equally infected by this fungus [19]. Apparently, scarce molecular evidence relating to different signaling cascades makes their exact taxonomic and phylogenetic classification very obscure [20,21]. The endemic geographical localization and strict restriction to the Alps, however, makes the fungus immune to the frigid cold pastures of the Tibetan Plateau and Nepalese Himalayas. Surprisingly, the available literature suggests that in addition to their natural habitats (soils), these species are also traced in the roots of plants [22]. Thus, the resurgence of interest warrants specific justification on the biological attributes of this invincible fungus.

**Figure 1:**
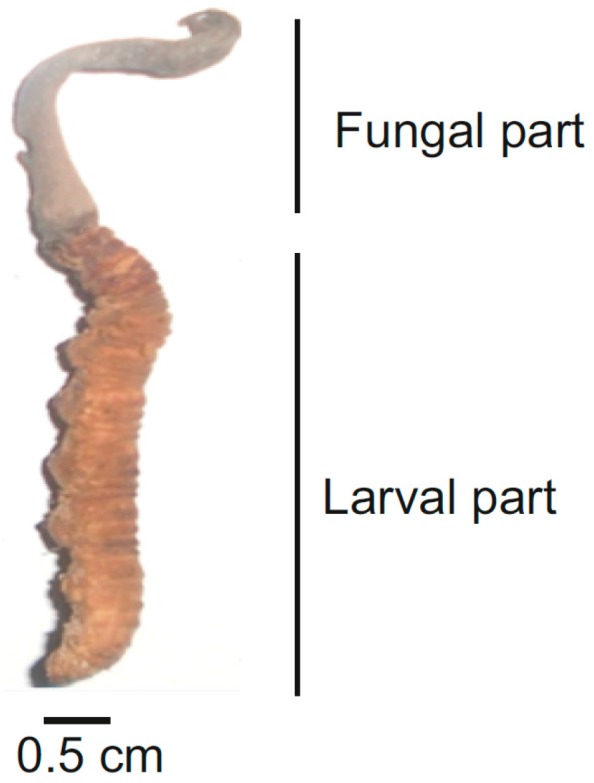
Morphology of *OS* (polished sample immediately after harvesting).

Structurally smaller, with a tiny blade-like fruiting body that blends like a camouflaged black stick into black soils, harvesting the organism is tedious, difficult and even expensive at times. The mature fruiting body of *OS*, however, is relatively bigger and can be harvested rapidly if traced (reviewed in [14]). Most harvesters reveal its difficulty as equivalent to browsing for a needle in a haystack. The stroma of the *OS* complex (fungus and insect larvae together) along with the mushroom tip (the fertile portion that gives rise to perithecia) is depicted in Figure 1.

*OS* is, in fact, a ruthless entomopathogen lurking in the Nepal Himalayas. Emerging evidence indicates different preferential modes of infection on juvenile larvae of the Himalayan bat moth *Hepialus armonicanus* [23], during which the fungus initially navigates larval weak body parts followed by penetrating the insects’ integument which is composed of chitin (a polysaccharide of *N*-acetyl glucosamine through 1–4 linkage). Thus, this fungus is endowed with the capacity to manipulate the behavioral patterns exhibited by the insects. Soon after, the rapid extension of mycelial networks and the subsequent dispensation of endotoxins to larval blood vessels take place. Owing to the tough nature of the cuticle (composed of wax and epicuticle), the fungus experiences a tough entrance through the cuticle and, hence, at times, the fungus encroaches host through its mouth and makes its way through the gut [24]. However, sufficient evidence is still lacking to validate this claim. Intriguingly, the cuticularized host epidermis affords protection to the host organism from drying out due to solar UV rays, preventing it from extreme desiccation plus other potential natural predators. Entomopathogens such as *B. bassiana* are endowed with mannitol-1-phosphate dehydrogenases and mannitol dehydrogenase which aid the organism in tolerating stressors such as H_2_O_2_, UV radiation and soaring heat by regulating the accumulation of mannitol. Extreme environmental stress thus makes these treasured herbs endowed with special bioactive compounds of enormous medicinal value, and this has been commercially exploited in Oriental medicine around the world for ages [25].

## 3. *Cordyceps* Ontogeny

Larval lethal infection by *Cordyceps*’ spores may occur at any stage of its development. Several cues seem to orchestrate the key processes in the fungal life cycle, viz. the mode of infection, subsequent growth, and asexual or sexual development. Biotrophic feeding behavior has been observed in *OS*, during which it initially colonizes insects’ larvae asymptotically, then switches to the nectrophic mode once the insect is dead, and thus it indicates that it is not an obligate biotroph but a facultative saprophyte [26]. Fungal species exhibit multiple host preferences, although they can also be strictly host-specific. The spores seek an opportunity to land onto the insects’ integument, become adhered to the surface, causing the initial binding interaction, and make their way in through the cuticle. Fluctuations of daytime and nighttime temperature in the high Himalayas force the vertical migration of larvae, causing the accidental introduction of larvae with fungal spores, and they get infected [27]. Intermittently, as mentioned earlier, spore may trespass en route to the mouth at the time when a larva starts feeding on the roots of small grasses (however, very scarce information is available to prove this).

Fungal entry into the insect hemolymph seems problematic because the fungus experiences difficulties as a result of the defenses provided by the larval immune system. An exchange of chemical signals facilitated by the receptors between the host and germinating fungal hyphae guides the right orientation for developing hyphae. Specific receptor-ligand and electrostatic mechanisms may be required for the proper attachment of the fungal cell to the cuticle, while this is not a unique event in *Cordyceps*. Success in establishment is achieved only when the fungus is able to find its compatible host, and, thus, partner selection proves to be an ideal step for fungal establishment, followed by specific substrate choices. The fungus responds against insects’ immune systems by invoking its own adaptive biochemical process and, thus, enhances the development of specific morphological structures [6]. Fungal entrance to the hemolymph suppresses the larval immune system and causes cessation of the insects’ lives, especially by starvation, convulsions or any physiological or biochemical disruption caused by the developing fungus [28]. Uncertainty still exists as to whether the life of infected larvae is hacked due to the active secretion of exogenous mycotoxins or due to the uptake of vital nutrients such as carbon and nitrogen by the rapidly extending fungus. The appearance of a germ-tube like structure soon takes place which facilitates in directing the fungus toward the interior of the cuticle rather than spreading horizontally merely in the cuticular layer [28]. Moreover, available ultra-structural and histo-chemical analysis reveals that larval infection occurs by a joint combination of enzymatic degradation plus mechanical pressure [28]. However, an exact mechanism on how mechanical pressure is exerted remains to be elucidated.

Following successful penetration, the conidia give rise to the appresorium and then to secondary hyphae during fungal entrance through the insect hemolymph, and extend further to the insects’ vital organs. Successive events after fungal establishment and colonization eventually cause the insect to get exhausted by losing vital body chemical constituents, leaving the insect to get paralyzed and completely mummified by the fungal mycelium in the long run [29]. In light of this, the detailed functional insights with a focus on the fungal pivotal role in attacking the larvae and the key components of the mitogen-activated protein kinase pathway and other pathways seem essential. Until now, very little information has been gained about the sexual and developmental processes in *Cordyceps* [18]. Owing to intensive extension of the fungal mycelium, the fungus gains maturity and starts to sporulate for its successive generations. Soon after the cadaver (mycosed pupae) is fully occupied by the fungal mycelium, the fungus exerts internal pressure, bursting out directly from the anterior part of the larvae (fronteal cortex), giving rise to a single-stem (rarely two) external structure called a mushroom. These events take place within the larval cadaver (an asexual stage). Rapid proliferation of the fungal mycelium within the cadaver causes the fungus to burst out in the form of a long-stalked fruiting body (teleomorph). In the insect hemolymph, the hyphal entrance and proliferation helps to breach the insects’ immune system by active secretion of a broad repertoire of secondary metabolites, such as cordycepin, adenosine, hydroxyethyl adenosine, dideoxyadenosine, etc. [14]. Also, extracellular enzymes with insecticidal properties (such as cyclic peptides, cordycepin, cepharosporolides, pyridine-2,6-dicarboxylic acid, 2-carboxymethyl-4-(30-hydroxybutyl) furan, dipicolinic acid, ophiocordin, etc. [30]) cause chemo-heterotrophic digestion of larvae and extensive fungal growth which may be seen protruding out of insects’ cadavers [31]. Although many enzymes are involved in fungal pathogenesis, very little is known about their characteristics, mode of action, regulation, cellular localization and the quantity produced. Sporulation occurs either asexually or sexually in sac-like structures called the ascus (six in each one). Apart from a few studies on *OS* anamorphs (*Metarhizium* sp., *Hirsutella sinensis* and *Beauveria* sp.), experiments aiming to elucidate the details of mycoattractants have not yet been conducted. Rapid exhaustion kills the ill-fated larva and it ultimately gets converted to a dead cadaver with an extensive proliferation of fungal mycelia. However, how long it actually takes for the fungus to kill the larvae once it is infected is still unclear.

The complexity in identifying *OS* on the basis of polymorphic DNA markers shows higher genetic variation between the *Cordyceps* population. Experiments revealed the isolation of many mitosporic fungi from *OS*, which were considered as asexual stages of this species [32,33], giving rise to an overwhelming question as to whether the *OS* complex differs from other species (mitosporic fungi) of endoparasiting insects [10]. This also raises questions as to whether the variable *OS*s assembled from different geographical niches possess different mitosporic fungi or could be classified as different species or sub-species [34]. *Hirsutella sinensis*, however, is only a correct anamorph (asexual stage) of *OS* localized in the Tibetan Plateau and shows a similar therapeutic potential to natural *OS* [10,35].

## 4. Events Incurred during Pathogenicity

### 4.1. Molecular Modality Displayed by OS

The underlying mechanism behind this rigorous process involves successive events of adhesion and recognition of host surface cues followed by direct penetration and colonization. As an example, hydrophobins such as *hsp1* and *hsp2* play vital roles in hydrophobicity, adhesion and virulence in *B. bassiana* [18]. The initial event of penetration involves conidial or ascospore assault through host recognition and adhesion, appressorium differentiation, generation of turgor pressure, secretion and release of different enzymes such as proteases and chitinases. The hydrophobicity conferred by the outer layer of the conidial wall which is comprised of well-organized fascicles of rodlets and the insect epicuticle mediates the adhesion process [36]. The colonization process involves adaptation to osmotic pressure, defenses that are acquired by cellular and humoral immune systems, acquirements of nutrients (carbon and nitrogen) and the late events that include budding and differentiation (germination within the insect cuticle, subsequent mycelial extension over insects, rapid multiplication and release of conidia for successive generations).

The formation of specialized infectious structures, such as appressoria or penetrant pegs, along with active secretion of secondary metabolites, facilitates the fungi to get over the quarry. For instance, in *M. anisopliae*, the cuticle-degrading enzymes such as *Pr1* (subtilisin protease) and dextrusins (toxic cyclic peptides) govern crucial roles during fungal pathogenicity [37]. In addition, adhesins *Mad1* and *Mad2* have a specific role in the adhesion of *M. anisopliae* to the insect cuticle [38]. The clustering of genes encoding secondary metabolites is highly conserved in *OS* and other insect pathogens [39]. The humoral reaction comprises the prompt secretion of various antimicrobial peptides (AMPs), and the activation of proteolytic cascades gives rise to melanization [40,41]. However, owing to the distinct infection mechanism of entomopathogens overcoming the hosts’ immunity, this process proves much more complex and intriguing at times. In addition, entomopathogens belonging to zygomycetes (such as Entomophthora, Zoophthora, Pandora, Entomophaga, and Erynia) are obligate parasites which are highly virulent and are very difficult to culture in laboratories, and thus prove very challenging to be used as biocontrol agents [42].

**Figure 2:**
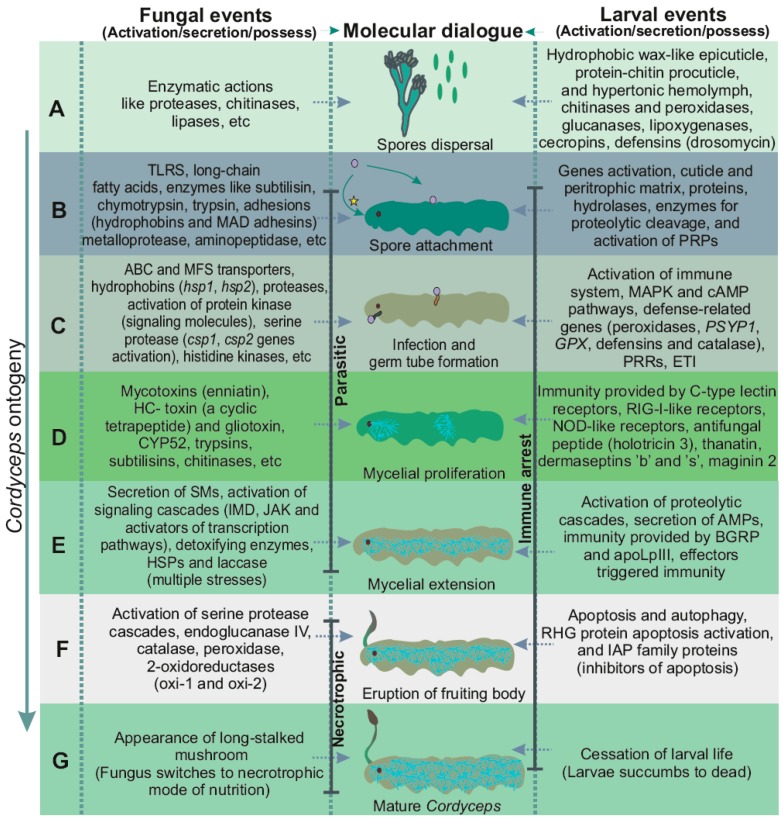
Fungal assault on insect larvae. (**A**,**B**) Spore landing and attachment: Sticky conidia are attached to larval cuticle by adhesins; (**C**) Infection and formation of germ tube: germination of conidia and extension of germ tube inside larval body; (**D**) Proliferation of mycelium: within larval body, hyphae extend continuously, giving rise to an extensive mycelial mass; (**E**) Extension of fungal mycelium: mycelium extends throughout larval body, colonizing every organ; (**F**) Eruption of fruiting body (ascocarp): soon after body gets colonized by fungal mycelium, it proliferates out from frontal cortex just between the eyes; (**G**) Mature *Cordyceps*: *Cordyceps* after erupting from larval head (larvae of Himalayan bat moth *Hepialus armonicanus).* * This entry-route of infection in *Cordyceps* is still unclear and thus requires further justification.

Entomopathogens belonging to oomycetes such as *Lagenidium giganteum*, *Aphanomyces laevis*, etc., are also pathogenic towards the larvae of mosquitoes [43,44]. For instance, *B. brassiana* (a broad-host-range entomopathogenic fungus) helps activate three immune-signaling cascades in mosquitoes (*Aedes aegypti*), viz. (i): Toll, Immune Deficiency (IMD), (ii): Janus kinase (JAK) signal transducer, and (iii): activator of transcription pathways [45,46]. Fungal infection activates Toll pathways, with the induction of serine protease cascades which culminates in Spatzle cleavage leading to transcriptional activation of several antimicrobial peptides along with other immune genes (Cecropin A, Defensin A and Serpin 27S) [47]. Surprisingly, *B. bassiana* is found to be a beneficial endophyte in plants (common bean, *Phaseolus vulgaris)*, in turn protecting plants from disease infections and herbivores [48]. Spores’ hygroscopic nature, and humid environments with frigid cold conditions cause stroma to crack open upon landing on insects’ integuments and gradually germinate and proliferate inside hosts’ bodies. The different associated processes and molecular interplay are elaborated in Figure 2. Apparently, most often the immune responses in insects are rapidly activated, which keeps fungal colonization at bay and this may include: (a) the production of antimicrobial compounds, lipids, proteins and secondary metabolites, (b) getting rid of the cuticle during development (molting), etc. As the cuticle represents a barrier between the fungus and larvae, the mechanism of action at the fungal-insect interface either leads to successful mycosis by the pathogen or successful defense by the host.

In the process involved, protein kinases (PKs), a major class of signaling molecules, play a crucial role in modulating the protein activity and gene expression in the fungus [49]. Though there is presently no detailed genomic, transcriptomic or complete kinome dataset on *OS* available in the public domain, a close relative, *O. polyrachis-furcata*, was sequenced in 2015 [50]. Recent transcripts profiling *Thitarodes jiachaensis* upon *OS* infection revealed the significant upregulation of 928 genes (58.70%), while the rest (653 genes) were downregulated [51]. Genes encoding cuticle and peritrophic matrix proteins, antimicrobial peptides, and enzymes responsible for proteolytic cleavage and pattern recognition receptors (PRRs) are likely to have pivotal roles during fungal infection [51]. While the infection process has been remarkably conserved, marked overlaps in the mode of nutrition and the underlying cellular signaling events, including the protein phosphorylation or dephosphorylation cycles, have been observed. The phylogenetically distant lying non-filamentous relative baker’s yeast (*Saccharomyces cerevisiae*) proves to be a well-studied model organism rendering functional insights about these MAPK (or ERK) and cAMP pathways [49]. Similar findings have also been observed in several filamentous fungi such as *Heterobasidion* sp*.*, *Laccaria* sp., etc. *OS*, however, endows several novel infective mechanisms used to parasite larva. Central to this are the involvement of transporters and other channeling systems of both the larvae and the fungus that shuttle elements and compounds across the membranes of both organisms. Molecular mechanisms for such parasitic roles may involve the active secretion of different enzymes and the role of major family transporters [ATP-binding cassette (ABC) transporters and Major facilitator superfamily (MFS) transporters]. ABC in fungi are involved in providing resistivity against drugs, and help provide defensive power against host-secreted secondary metabolites [52]; however, MFS are involved in the transport of a wide range of substrates and are considered to be involved in nutrient sensing. Moreover, restricted by a narrow lifestyle behavior, this fungus (*OS*) possesses more than two-fold fewer dehydrogenase-encoding genes than other entomopathogens (103 vs. 237) [39]. In addition, entomopathogens are endowed with some more amino acid and peptide transporters than other fungi, which allow them to access a range of protein degradation products from the insect sources. Entomopathogens such as *M. robertsii* and *B. bassiana* possess a similar repertoire of MFS transporters; however, *B. bassiana* have more ABC transporters compared to a previous one (75 vs. 55 respectively).

The genome-wide inventory could prove that the reduced number of gene families that encode cytochrome P450 enzymes (alkanes and fatty acids are substrates for these specific subsets), chitinases and cuticle-degrading proteases in *OS* compared to other insect pathogens suggests the preferential mode of infection of *OS* through mouth parts rather than the cuticle [24]. The physical barriers afforded by the cuticle and peritrophic membrane are breached by a broad repertoire of enzymatic arsenals of conidia. Enzymatic degradation is enforced by the possession of different extracellular enzymes or other enzymes that concentrate within the host-cell membrane. Defense-related genes in host larvae encode peroxidases, class III peroxidase (PSYP1), glutathione peroxidase (GPX), defensins and catalase. Moreover, the genes responsive for defense in fungus are endoglucanase IV, catalase, peroxidase, and two oxidoreductases (oxi-1 and oxi-2) [53]. However, a clear mechanism for the mode of action of these enzymes and the process of infectivity is still obscure and worth investigating.

A precise signaling mechanism facilitated by the presence of effectors and receptors at the host-pathogen interface helps the spores to adhere onto the larval cuticular layer. The fungal secreted effector molecules possess a varying capability to modulate the physiology of their hosts. Toll-like receptors (TLRs) play a central role in recognizing host cells and the subsequent responses to the microbial pathogens [54]. Moreover, recent identification of other non-TLR pattern recognition receptors (PRRs) such as C-type lectin receptors, RIG-I-like receptors, and NOD-like receptors helps to shed light on the complexity of innate immunity [54]. The PRRs such as β-1,3-glucan recognition protein and apolipophorin III (apoLp-III) in *Thitarodes pui* confer immune response to *B. bassiana* infection [55]. The role of effectors is to localize the fungus onto the insects’ bodies. Effector-triggered immunity (ETI) may help protect the insect from host-adapted biotrophic fungal pathogens. Unlike in other organisms, ETI in *Cordyceps* is rarely followed/associated with a hypersensitive response (HR) with programmed cell death (PCD) at the infection site. Moreover, two different types of PCDs (apoptosis and autophagy) required for the completion of metamorphosis have been recorded in different tissues of the insects [56]. Some insects (such as *Drosophila melanogaster*) possess RHG (Reaper, Hid and Grim) protein apoptosis activators and IAP family proteins (inhibitors of apoptosis) with roles in ubiquitination in the muscle PCD of insects [56]. Owing to the successful landing of spores on to the cuticular surfaces, modification of insects’ integuments takes place ,followed by the germination of spores [28], and the adhesion of germlings. *OS* possess special compounds with insecticidal properties (such as short-chain fatty acids) which serve as arsenals against the barrier afforded by insect integuments and help dissolve the cuticular layer [36]. Furthermore, spore germination is accelerated by the possession of several enzymes that help in breaching the host barrier, simultaneously nourishing germinating spores.

**Figure 3:**
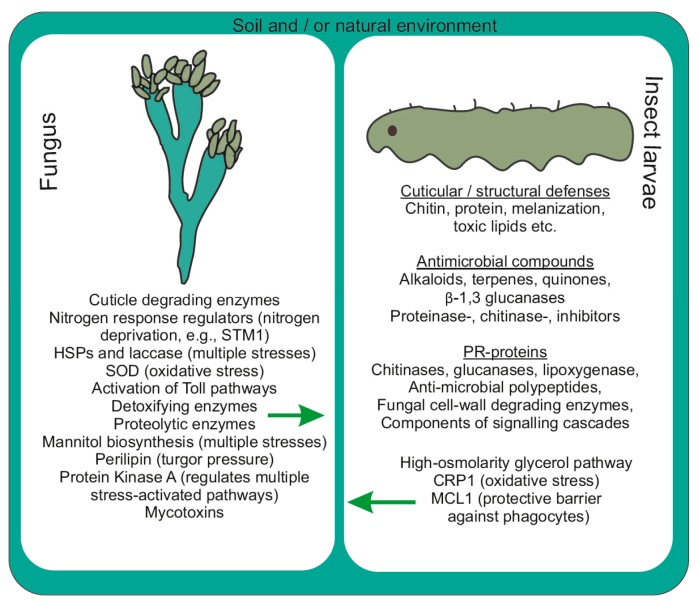
Defensive munitions observed during fungus-insect interactions (molecular interplay between two organisms).

The larval integument comprises chitin, proteins, lipids, various enzymes and phenolic compounds [57]. Enzymes at larval integuments include subtilisin, chymotrypsin, trypsin, metalloprotease, aminopeptidase, post proline dipeptidyl peptidase, post alanine peptidase, serine carboxypeptidase and zinc carboxypeptidase [58]. During fungal establishment within larvae, enzymes such as esterase and proteolytic enzymes (aminopeptidase, endoprotease and carboxypeptidase) are secreted initially, followed by the production of chitinase and lipase at later periods (after few days) of infection. Subsequently, cuticle-degrading enzymes such as serine-protease (with fibrinolytic activity) encoded by *csp1* and *csp2* genes help to disintegrate the larval cuticular layer and facilitate fungal establishment onto the larval surface (Figure 3) [59].

Studies on *Metarhizium* (an anamorph of *Cordyceps*) infectivity depict that the fungus needs to trespass components of the insect cell membrane which includes the hydrophobic wax-rich epicuticle, the protein-chitin procuticle, and the hypertonic hemolymph [60]. Additionally, the conidia of *OS*, *M. anisopliae* and *B. bassiana* possess enzymes such as proteases, chitinases and lipases, which makes them more proficient in dissolving insects’ integuments and at the same time nourishing the germinating conidia [61]. These enzymes are broadly shared by fungal pathogens, both by phytopathogens and entomopathogens. Genes encoding secreted proteolytic enzymes in *C. militaris* harbor altogether 61 families, with most included in the serine proteases and metallopeptidases families [18]. Most of these genes are involved in the pathogenicity of *C. militaris*. Other proteolytic enzymes include a family of trypsin-like proteases such as endoproteases, exoproteases, metalloproteinases, several aminopeptidases, carboxypeptidase A, lipase, esterase, chitinase and *N*-acetylglucosaminidase [58]. Among these, endoproteases endow productive roles in most species. Intriguingly, the active secretion of only proteolytic enzymes may not be the sole fungal pathogenicity factor acting as an entomopathogen, but still these enzymes could be considered as the secondary determinants of basic compatibility factors [58]. Transcriptional activation of pathogenic genes in *OS* by solar radiation seems rational; however, sufficient information is needed to validate this claim. In addition, intense solar UV radiation at higher elevations might prove to be lethal for both the fungus and its conidia, ultimately limiting their life cycle. Apart from this, several enzymes such as Cu–Zn supermutase and peroxidases afford protection to the fungus against harmful radiation and ultimately prevent against the generation and outburst of reactive oxygen species [62]. In *Metarhizium*, the induction of RNA-binding proteins, photolyase and heat-shock protein allows the fungus to endure tolerance to cold stresses, UV and several other stresses (heat and osmotic pressure).

The intricate combination of mechanical pressure (exerted by infection structures (appressoria and penetration pegs)) plus cuticle-degrading enzymes such as serine-proteases breaches the larval cuticle [59,60]. In addition, *OS* gene families encoding cytochrome p450 (CYP) sub-family CYP52 enzymes (for metabolism of insect epicuticular lipids), cuticle degrading proteases (two trypsins), 17 different subtilisins and nine different chitinases found in *OS* ensures successful fungal infection of their hosts [63]. Surprisingly, protein families involved in adhesion to insect cuticles and the formation of appresoria are reduced in *OS* [63], suggesting the evolutionary removal of redundant genes that encode proteins for adhesion. This could be viewed as a fungal adaptive strategy to reside in harsh environmental conditions and also to preserve their energy by repressing their cryptic and silent gene clusters. Moreover, an available hypothesis suggests that the conidium undergoes a stereotypical series of changes, producing a swollen hyphal tip that gives rise to a stick holdfast (appressorium) as is observed in *Metarhizium* [64].

### 4.2. Antifungal Peptides in Insects: A Component of Defense Munitions

All insects are susceptible to fungal infections, but very occasionally do they contract viral and bacterial diseases. Insects’ larvae customize an entire battery of defenses, eventually activating their immune system to cope with highly bioactive fungal enzymes. Fungal infective arsenals produced by the active secretion of secondary metabolites (SMs) are programmed by the expression of endogenous genes, which ultimately breach the structural and cuticle barrier of insects. Cases of the active secretion of the insect-specific immune suppressants have also been traced in some fungi. However, larvae of host insects are endowed with an excellent surveillance system that enables the recognition of threats, signal transduction and the presence of other pathogen in their vicinity. The activation of several pathogenesis-related (PR) proteins has been identified with increased activity levels of chitinases and peroxidases during fungal infection in some aphids such as *Smynthurodes betae* and *Slavum wertheimae* [65]. The identification of 19 PR proteins families (such as chitinases, glucanases, lipoxygenase, fungal cell-wall degrading enzymes, etc.) endowed with anti-fungal and anti-insect activities has been achieved so far. The defense metabolites involve the active secretion of low-molecular-weight compounds, antimicrobial peptides and proteins that are antagonist towards fungus [66]. These SMs, along with antimicrobial peptides, are involved in either constitutive or induced resistance to act against the infective fungus; however, for the *OS* to get established within the larval body, the fungus needs to breach these larval secretions within the body. Nonetheless, the paucity of adequate knowledge on the mechanism of interactions between the larvae and fungus makes difficult the development of novel antiviral strategies.

Defensins in neutrophils of some animals such as rabbits, rats and humans have antagonistic properties against the conidia and hyphae of *Candida albicans* and *Coccidiodes immitis* [66,67]. Successful studies on insect-derived antimicrobial peptides, such as cecropins, a linear peptide found in the hemolymph of the giant silk moth *Hyalopora cecropia*, have been conducted [68]. The critical dosage of cecropins has been found to be 25 to 100 µg/mL, and it has ability to suppress the growth of *Aspergillus* species. However, the relatively low dosage of cecropin A (12.4 µg/mL) obtained from *H. cecropia* is sufficient to suppress the growth of *Fusarium moniliforme* and *F. oxysporium*, while cecropin B (9.5 µg/mL) suppresses *A. fumigatus* [69]. Similarly, *Drosophila melanogaster* is endowed with an insect defensin called drosomycin, which is very effective against *F. oxysporum* [70] having minimum inhibitory concentration (MIC) of 5.9–12.3 µg/mL. Moreover, the hemolymph of *Sacrophaga peregrina* and *Holotrichia diomphalia* is endowed with an antifungal peptide called holotricin 3 which has a pivotal role in checking the growth of *C. albicans* [71]. Similarly, thanatin, a non-hemolytic antimicrobial peptide with a lethal role against *F. oxysporum* and *A. fumigates*, is produced by *Podisus maculiventris* MIC at a concentration of 24–48 µg/mL [72]. Moreover, dermaseptins “b” and “s”, obtained from *P. sauvagii*, are effective against *C. neoformans* at the critical dose range of 60.0 and 5.0 µg/mL, respectively. Again, magainin 2, found in *Xenopus laevis*, is effective against *C. albicans* at a concentration of 80 µg/mL [73]. Recently, a review paper on insect-derived antifungal peptides was published, revealing the antagonistic properties of different peptides against fungal development, and the highest activity was observed in the final instar larvae [74].

### 4.3. G-Protein–Coupled Receptors (GPCRs) in Fungus and Insects

The fungal infection cycle in insects undergoes a complicated process that involves several major steps, such as appressorium formation, the emergence of penetration pegs required for cuticle penetration, trespassing the epidermis, blastospore formation and rapid spread throughout the hemocoel. Recognition of host-based transcriptional analysis during fungal pathogenicity has gained increasing interest these days for most fungal species as it facilitates in identifying the key virulent genes responsible for fungal infection. Unlike in several other phytopathogens, entomopathogens render an immediate response with fluctuations in nutritional status, osmolarity stresses and host defense systems. Thus, the search for signal transducing receptor proteins seems crucial. However, owing to the lack of adequate information on GPCR (a transmembrane receptor) in *OS*, other ascomycete entomopathogens such as *M. anisopliae*, *M. acridum* and *B. brassiana* might prove to be excellent models for elucidating an exact mechanism of stress response signaling and virulence properties. Available evidence on these pathogens suggests that GPCR plays a crucial role in the transduction of the signaling cascades between the host and pathogen that are encoded by several genes in entomopathogens. Moreover, GPCR is a heterotrimeric receptor with α, β, and γ subunits, among which the α-subunit is thoroughly studied because of its profound importance in pathogenicity to different organisms (insects, plants, etc.). Surprisingly, unlike the commonly observed heterotrimeric GPCR in other eukaryotes including fungus, identification of fourth subunit of GPCR in the genome of *Metarhizium* and some plant pathogens such as *Stagonospora nodorum* and *Ustilago maydis* has been revealed with still-unknown functional roles but with a profound chance of being involved in the pathogenicity of animals and plants [75,76]. GPCR regulates diverse activities by the specific recognition of ligands at the cell surface, as is observed in *M. acridum* which transcribes the activation of GPCR on the exoskeleton of cockroaches [9]. Thus, it is very necessary to study the evolutionary aspects of GPCRs and their signaling pathways, as they may help us to shed light on the insect-specific features. This may help us develop new insights, which may result in elucidating novel methods along with wider practical applications. However, I believe that it is too early to provide any general scheme or outline about the mechanism of GPCRs in this *OS* larval-fungal complex before the complete picture emerges.

### 4.4. MAP Kinase Cascades: Regulation of Transcription

**Figure 4:**
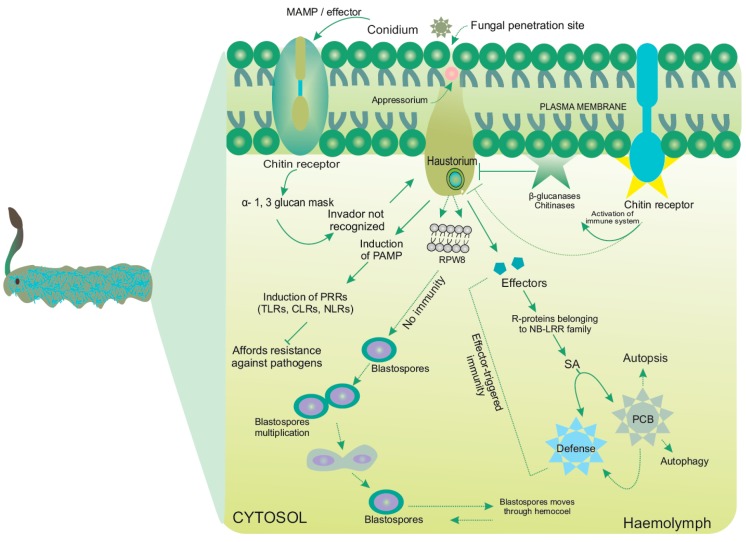
Detailed mechanism of fungal assault to insect larvae. Unlike in mammals, fungal cell-wall is endowed with several glycans, glycolipids and proteins. PAMPs trigger upregulation of immunity via several pathogen-recognition receptors (PRRs), such as Toll-like receptors (TLRs), C-type lectin receptors (CLRs) and NOD-like receptors (NLRs). CLRs such as N-linked mannans, galactomannans, β-1,3 glucans, α-mannals and α-mannosyl residues are detected by MR, DC-SIGN, Dectin 1, Dectin 2 and MINCLE, respectively. RPW8: Atypical resistance protein. DC-SIGN: Dendritic cell-specific ICAM3-grabbing non-integrin, MAMP: Microbes-associated molecular pattern, MINCLE: Macrophage-inducible C-type lectin, MR: Mannose receptor, NB-LRR: Nucleotide binding leucine-rich repeat domain, NOD: Nucleotide-binding oligomerization domain-containing protein, PAMP: Pathogen-associated molecular pattern, PCB: Polychlorinated biphenyls, SA: Salicylic Acid [36,56,80]. Solid and dotted arrows represent detail molecular mechanism in most entomopathogens and *Cordyceps* sp. respectively, while T-arrows represent resistance mechanism of the insects against the invading pathogens.

Mitogen-activated protein kinase (MAPK) (originally extracellular signal-regulated kinases) and cAMP (cyclic adenosine monophosphate)-PKA (protein kinase A) cascades are the key players in pathogenicity and disease development in most fungi including *OS*. Diverse key molecules are secreted during this process, which signal membrane proteins and receptors, causing transcription factors to activate several genes. Moreover, the MAPK cascades govern a variety of cellular responses, ranging from proliferation and differentiation to stress adaptation and PCD. A transcript profiling study during fruiting in *C. militaris* suggests the activation of Zn_2_Cys_6_-type transcriptional factors and the MAPK pathway; however, the PKA pathway seems to not be induced [18]. Different cross-talk in the signaling pathways has been observed in *OS*; however, no evidence suggests the pathogenic role of *OS* in plants as is observed in *Metarhizium* species, shown in Figure 4 [77,78]. In *B. bassiana*, Bbslt2 MAP kinase governs control over the fungal growth, conidiation, cell wall integrity, and virulence [79].

The role of MAPKs in exchanging signals provides molecular guidance to the development and differentiation process [81]. A few of them, such as *FUS3/KSS1, HOG1*, etc., have proved vital in shedding light on fungal pathogenicity. Among very scarce studies relating to MAPK pathways in *OS*, the close relative *B. bassiana* is now known to possess five different genetic regulatory cascades of MAPK (*FUS3*, *KSS1*, *HOG1*, *SLT2/Mpk1*, and *SMK1*) with their respective involvement in the sensing of pheromones (mating), the growth of filaments, responses to high osmolarity, cell wall integrity, and ascospore formation [82,83]. Genome sequencing revealed the presence of both *MAT-1* and *MAT-2* expression in vegetative mycelia, which possibly indicates it to be homothallic and it reproduces by selfing [39,84]. Moreover, in *M. acridum*, the *MaMK1* gene which encodes the FUS3/KSS1-type MAPK of the YERK1 sub-family in yeast is essential for appressorium formation and also for successful adhesion and penetration of the insect cuticle reaching into the hemocele [85]. In *B. bassiana*, the virulence of the Bb*mpk1* gene encoding a YERK1 sub-family MAPK to host insects provides significant improvements in the adhesion to the larval cuticle and the successive development of appressoria [86]. Studies on Bb*hog1*, a MAPK-encoding gene in the same species, reveal it to have a conserved function of HOG1 MAPKs in regulating responses towards abiotic stress [87]. The knock-out mutants (ΔBb*hog1*), however, tend to have reduced virulence to insects, either due to low viability of spores or decreased capability of spore adherence to the insects’ cuticle [87]. Also, in *M. acridum*, a HOG1-type MAPK gene named Ma*HOG1* was found to have a crucial role in affording fungal tolerance to stress and virulence to insects [85]. In *M. robertsii*, protein kinase A helps in regulating the control expression of several virulent factors [88], while a protein named “Perilipin” helps combat against osmotic stress, and regulates lipolysis and the formation of infection structures [38]. Similarly, several phytopathogenic fungi such as *Neurospora crassa*, *Magnaporthe grisea*, *Colletotrichum lagenarium,* and human pathogenic fungi such as *C. albicans* are endowed with a similar MAPK cascade that signals in adapting the osmotic stress and heat shock proteins with less ability to adapt to conditions of high osmolarity [89,90,91,92]. Thus, targeted functional studies with a focus on HOG1 homologs in *OS* could help us in revealing the functionality of this signaling cascade in the host-pathogen system. Even though these all suggest that MAPK has a significant role in fungal pathogenicity, still the insect-specific adhesion factor needs to be well studied in *OS*. Adhesins such as MAD1 and MAD2 in *M. robertsii* help the fungus to adhere to the larval cuticle and plant epidermis, respectively, suggesting the upregulation of different genes according to its distinct lifestyle [38]. The fungal cell wall comprises chitin, (1–3) β-d-glucan, (1,6) β-glucans, lipids, and peptides embedded in a protein matrix [93]. Remodeling the insect cell surface and evasion of the immune response in the fungus is mediated by several proteins. For instance, MAD1 and MAD2 proteins help *M. anisopliae* to attach and adhere to the surface of both plants and insects, while in *S. cerevisiae*, MAD1 and MAD2 proteins help it adhere to the insect cuticle and plant surface, respectively [38].

### 4.5. Histidine Kinases: Osmosensing and Beyond

Fungi, being extremophiles, cope very efficiently even in the most challenging conditions of varying stressors, including nutrients, abiotic or biotic factors. The optimum temperature range for the growth of entomopathogens such as *Metarhizium* and *Beauveria* is 22–26 ^°^C; however, most other fungal species grow either above 32 ^°^C or below 10 ^°^C [94,95]. In addition, having a very complicated and narrow lifestyle, *OS* exhibits a strict and inflexible metabolism with growth restricted to a very low oxygen concentration, which is host-specific and also very selective with substrate choices. The UV rays of solar radiation and direct heat impose profound effects in the viability of the conidia, affecting its successful colonization and rapid multiplication in natural conditions [96]. Transmembrane proteins such as histidine kinase (e.g., PHY1p in *B. brassiana*) play vital roles in signal transduction across the cellular membrane. Higher gene expression of CmHK in *C. militaris* was recorded after 16 h of fungal mycelium exposure to blue light, which then plummeted down. This observed characteristic of CmHK in *C. militaris* corresponds to the characteristics of histidine kinase [97].

## 5. Enzymes Involved in Fruiting Body Production

Ubiquitin-like activating enzyme E1 controls the production of the fruiting body in *C. militaris* [98]. Repression of the *Cmwc-1* gene (a homolog of the blue-light receptor gene *white collar-1 (wc-1)* in *N. crassa*) results in the formation of thicker aerial hyphae, the development of a disordered fruiting body, and a reduction in the formation of conidia, cordycepin and carotenoid production in *C. militaris* [99]. However, the resumption of functional properties and characteristics of knock-out mutants was observed upon hybridization of these with the wild strains of the opposite mating type [99]. *C. militaris* in in vitro conditions revealed the involvement of different enzymes (cytochrome oxidase subunit I, ubiquitin-like activating enzyme) in the formation of the fruiting body, while the serine/threonine phosphatase encoding gene was involved in in vivo conditions [98]. Also, an enzyme with fibrinolytic activity (chymotrypsin-like serine metalloprotease), having a potential role of developing therapeutic agents for thrombosis, has been extracted from *C. militaris* [100].

## 6. Genomic Information

The whole genome shortgun (ANOV00000000.1) revealed the genome size of *OS* to be 78.52 Mb with 6972 protein-coding genes in total [50]. A search for *OS* in GenBank revealed 29,647 entries, most of which were sequences that code for ribosomal RNA, apart from some sequences (at least 184) with accession numbers which include: AY195841.1, EU282383.1 and EF495094.1 (NCBI online database last accessed 15 October 2016). These 184 sequences (entries) for serine-protease enzymes in *OS* are encoded by the genes *csp1*, *csp2* and *Pr1* (NCBI online database last accessed 15 October 2016). The whole genome sequence of a closely related species, *Cordyceps militaris* (32.2 Mb genome size), has been available to the public since 2011 [18]. The genome size of *O. polyrhachis-furcata* (strain BCC54312) is 43 Mb, with 6793 protein-coding genes, which is similar to *OS*, while substantially less than *M. robertsii*, *M. acridum*, *B. bassiana*, and *C. militaris*. The assembled genome has been deposited as an NCBI Whole Genome Shotgun (WGS) project under accession number LKCN00000000 and the data of the sequenced samples were deposited at the NCBI BioSample database under the accession number SAMN04099149.

The genome size of *C. militaris* is smaller when compared to its anamorph with a broad-host-range species such as *M. anisopliae* (39.0 Mb) or the locust-specific *M. acridum* (38.0 Mb) [9,63]. In addition, the protein-encoding genes for this *Cordyceps* species (ca. 9684 protein genes) are also fewer than those of the anamorph species (10,582 and 9849 protein genes for *M. anisoplliae* and *M. acridum*, respectively) [9,18]. Apparently, the genes with putative secreted proteins are higher for these three species (15.9%, 17.6% and 15.1% for *C. militaris*, *M. anisopliae* and *M. acridum*, respectively) compared to other ascomycetes (5.10%) whose whole genome has been sequenced [9,18]. Genes encoding mycotoxins were not found in *C. militaris*, making this a unique species for human use [18]. Thus, the comparative genomics among *C. militaris*, *M. anisopliae* and *M. acridum* show the highly reduced amount of genes in *C. militaris* in each category that involves virulence/detoxification, transportation, protein synthesis, signal transduction, homeostasis, etc. However, the species-specific genes were found to be higher in *C. militaris* [18].

## 7. In Vitro Fungal Culture and Characteristics

Different strains of *Cordyceps* sp. harbored in different geo-environments produce variable cultural characteristics with varying therapeutic properties [101]. The size of the colony, shape and color may vary widely with different strains when observed on artificial solid media and they do not vary with age [30]. Laboratory cultivation of *OS* is difficult, while the cultivation of other species of *Cordyceps* such as *C. militaris* has been tremendously successful [102,103]. The major bottleneck for the artificial cultivation of *OS* is the low fungal infection rate of artificially inoculated insects [59,104]. In some extreme conditions, when successful inoculation is achieved, the mycelium of *OS* can be grown to about a 1 cm diameter after two weeks [103]. Even though difficulties in growing *OS* in artificial media persist, an increasing number of pseudo-*Cordyceps* products derived from mycelial cultures of anamorphic forms of these fungi have become commercially available [105]. To meet an increasing demand and to diminish the prevailing huge pressure on natural resources, mass cultivation of living strains of *Cordyceps* has been successfully performed at industrial levels using bioreactor technology [106]. The cultured mycelium of *OS* is transparent to translucent, in an aggregation, mostly in irregular blocks [107]. Most often, difficulty in identifying actual relationships between *Cordyceps* anamorphs and species persists because some species produce anamorphs readily in nature but not in culture [108]. However, this could now be circumvented using robust molecular biology approaches. Although natural *OS* specimens harbor significant pharmaceutical properties that have been used in traditional Chinese medicine for many years, the commercial cultivation of this fungus on moth larvae to produce fruiting bodies has not been successful so far. 

Fungi other than *OS* originating from natural *OS* specimens could be an important asset for developing alternative products of this natural specimen and may significantly contribute to the growth and cultivation as well as the pharmaceutical effect of natural *OS* specimens. *Hirsutella sinensis* is the only known anamorph of *OS*, and it is grown widely in controlled conditions, is a potential source of broad therapeutic agents and harbors the same clinical properties along with less associated toxicity than the natural *Cordyceps* [109,110,111]. However, the in vitro culture of *OS* easily gets infected with diseases and natural pests [112]. In addition, doubts and difficulties persist in verifying whether an identical assortment of bioactive ingredients exists in the fruiting bodies and cultured mycelia in synthetic laboratory conditions.

Available literature suggests that *Cordyceps* can be cultured on potato dextrose agar plates for 96 h at 28 ^°^C and can also be reassigned to nutrient broth containing (g/L): peptone (10), glucose (30), V_B1_ (0.05), KH_2_PO_4_ (1), MgSO_4_ (0.2), at pH 6.0 with shaking at 120 rpm for 96 h at 28 ^°^C [113,114]. Supplementary substrates such as rye grain, millet or rice could be added or replaced, while rye or millet substrates represent a better choice and generate a superior quality product than rice. Even a higher quality product can be achieved using silk worm residue from dead silk worms as a substrate. The obtained mycelia can then be harvested, lyophilized and stored at −80 ^°^C until further use [115]. The inoculums for fungal culture could be prepared with ascospores or fungal hyphae, which are asexual stages of fungus which eventually give rise to asexual fungal hyphae. However, no success has been achieved so far for developing sexual samples of *OS* from artificial cultivation. Fruiting bodies are frequently employed for investigating their usefulness. Recently, research has concentrated on studies of cultured mycelia, a substitute for natural *Cordyceps*, so as to diminish pressure on natural resources. Moreover, mechanism-based technological improvements with disease-oriented pharmacological studies are required to guarantee their clinical application and integration into daily consumables.

It is now widely known that the 80%–85% of the medicinal mushroom products are extracted from the fungal fruiting bodies, and the remaining ones are derived from the mycelium culture [116]. Various studies and experiments have been performed in order to culture the fungus in the insect larvae (pupae of silkworm *Bombyx mori*) and various cereal grains, and success, to certain degree, has been achieved in both [117]. However, very little success is achieved when the fungus is left to grow on the insect larvae. *Cordyceps* grown in silkworm residue or cereal grains as a substrate and the natural ones share the same range of medicinal properties [118].

## 8. Secondary Metabolites Repertoire in *OS*

*OS* secretes a broad repertoire of secondary metabolites (internally and externally) with the potential to breach the larval cuticular layer and to consume the larvae very effectively. These secreted secondary metabolites both in solid surface media and in the axenic culture system provide the fungus with enhanced and superb medicinal properties. Extracellular polysaccharides (exopolysaccharides) are the most abundant compounds that are found in the fungal culture. Available studies to date have mostly been focused on the pharmaceutical products of this fungus (reviewed in [119]). Overall, the fungus is known for its medicinal uses as the “Himalayan Viagra”, for its tremendous capability to stimulate the sexual desire of men. Besides these uses, it has been exploited for its efficacy in the production of several drugs with huge therapeutic implications.

### Chemical Constituents, Bioactive Compounds and Uses of Natural Cordyceps

The increasing resistivity of pathogens towards antibiotics has become an extreme health problem throughout the globe. With constant and continuous exposure to antibiotics, pathogenic microbes have evolved resistance to single or multiple traditional antibiotics [49,120]. Furthermore, the aggravating losses in agricultural products by farm pests necessitate further studies on new bioactive products with potential biocontrol capabilities.

Chemical profiling of *OS* has gained increasing interest owing to its special and distinct properties. A cocktail of this fungus-host entity (a complex of *OS*) has been widely administered orally along with food and water as *ad libitum*. The fungus can be consumed as a stuffing in chickens, old ducks, hen’s meat, or as a ground soup to treat patients suffering from cancer and asthenia [121]. Additionally, it can also be consumed by cooking it with pork, sparrow and turtle, and used in treating fatigue, male impotence, and hypo-sexuality [121]. A traditional dosage of wild *Cordyceps* ranges from 3–4 g/day, sometimes as high as 9–10 g/day. A critical care dosage may scale up to a range as high as 30–50 g/day [122]; however, the actual dosage depends on the type of substrates used and the age of the fungus. Intriguingly, the stage at which the fungus acquires its highest medicinal activity is still ambiguous. Implications have been made that more active components are integrated within the fungus during the process of killing the insect and consumption as a whole. The fruiting body (fungal partner) is said to inherit similar chemicals with the same invigorating bioactivity because of the gradual replacement of insects’ constituents with fungal mycelium [123]; however, sufficient scientific evidence is still unavailable to validate this claim. Moreover, with no mycotoxins associated with this fungus, this has received increasing attention recently.

Due to various in-built pharmaceutical chattels, members of *Cordyceps* species have multiple and wide-ranging uses in traditional Chinese medicine [124]. As the caterpillar is completely invaded by *OS* mycelium and is eventually consumed, the two parts (fungus and larvae) are thought to share similar chemical constituents with a similar range of pharmacological functions [123]. Most often, the whole fungus and also the secondary metabolites secreted by fungus are consumed. Due to its sweet and slightly astringent taste [1], along with an oily quality, this species complex is considered to acquire higher nutritional properties [118]. Bioactive compounds such as cordycepin (3’-deoxyadenosine) and d-mannitol [125], ergosterol (free ergosterol and esterified ergosterol) [126], polysaccharides, glycoprotein and peptides [25] are found engraved in this organism. Genes that encode glucose–methanol–choline oxidoreductase and telomerase reverse transcriptase are involved in cordycepin (an immunosuppressive agent) formation in *C. militaris* [98]. Other major components include saccharides and sugar derivatives (for instance d-mannitol), sterols, superoxide disproportionation enzymes, polysaccharides (3%–8% of total weight) [127], including cyclofurans, β-glucans, and β-mannans, complex polysaccharides [128], anti-tumor adenosine derivatives, ophiocordin and l-tryptophan [129]. Vital chemical components in *OS* include cordycepic acid, glutamic acid, amino acids (phenylalanine, proline, histidine, valine, oxyvaline, arginine, l-tryptophan, l-arginine, and Lysine), polyamines (1,3-diaminopropane cadaverine, spermidine, spermine, and putrescine), cyclic dipeptides, saccharides and sugar derivatives, sterols [130,131], nucleotides and nucleosides (adenine, uracil, uridine, guanine, guanosine, thymidine, deoxyuridine and cordycepin), 28 different types of saturated and unsaturated fatty acids, their derivatives and other organic acids (oleic, linoleic, palmitic and stearic). Some vitamins traced in this fungus include B1, B2, B12, E and K. It also harbors alkaloids and fatty acids (mainly oleic, linoleic, palmitic, and stearic acids) [2,132].

*Cordyceps* has attracted profound research interest for its anti-oxidant activity with substantial evidence to support its use in the treatment of a wide range of diseases [30], and for the replenishment of physical health [133]. It possesses anti-viral activity, affects human leukemia [134], has immuno-modulating, cholesterol-reducing, anti-oxidant effects [135], stabilizes the blood sugar metabolism [136] and has profound potential to increase stamina and libido [137,138]. Sterols help in preventing chronic kidney inflammation, lupus erythematosus and asthma, and increase the contractile ability of the atrium and ventricle [139]. The caterpillar fungus has a hypoglycemic effect and may be beneficial for people with insulin resistance [140]. Owing to its diuretic effect, it is crucial for the prevention of nephralgia [141]. Additional attributes include the potential to adjust protein metabolism, inhibit lung carcinoma, replenish kidneys, attenuate renal fibrosis [124], soothe lungs [142], treat aging disorders [143], stimulate the production of corticosteroids [144] and improve blood circulation [1]. This aids in increasing superoxide dismutase (which functions as a free radical scavenger, reduces oxidation and aging of cells, and has anti-inflammation effects) activity by reducing the formation of free radicals [145]. The fungus also delays cognitive decline [135,139], thus endowing a protective effect against ischemia-induced brain infarction by modulating 17β-estradiol production [114]. The increase of ATP levels in the body by almost 28% has been observed with the use of this fungus. This highly treasured herb is being used for treating chronic coughs, chronic bronchitis, insomnia, and hypertension, and it provides endurance, vitality and longevity [146]. It is also used in the treatment of pulmonary emphysema, anemia, night sweats [142], and for mycocardial mitochondrial ATP generation [147,148] and as a health booster. Other conditions treated with the fungus include: debilitating asthma, respiratory diseases [142], consumptive coughs with hematemesis, arrhythmias [149], impotence, spermaturia, lumbar and knee pains, hypocholesterolemy [150], hypoglycemic activity in genetically diabetic mice [129], high blood-lipid levels [151] and it also improves the internal balance mechanism and is also used as a preventive remedy against group A streptococcal infection [152]. The aqueous extract may help prevent tumor metastasis in mice as an adjuvant agent in cancer chemotherapy [153] and it also cures bone marrow and intestinal injuries [154].

## 9. Challenges and Opportunities

Extreme harvesting of this fascinating species before or after maturity poses a big risk which could lead to the rapid decline of natural specimens. Harvesting of *Cordyceps* prior to spores’ maturity and dispersal results in a progressive reduction in the number of the spore population, along with decreasing the rate of host-insect interactions. In addition, global climatic fluctuations have a profound impact and negative consequences on the life cycle of *OS*, leading to a sharp decrease in the *Cordyceps* population. Furthermore, a chunk of harvesters migrating at once to the collection sites during harvesting may damage young moths or the young *Cordyceps*, subsequently causing a rapid decline and deteriorating reduction in the *Cordyceps* numbers. Nevertheless, flouting the general rules set up by the local government, a lack of proper policies on pastoral management, and flocks of grazing sheep that uproot most plants while grazing at higher elevation affect the alpine grassland ecosystem and certainly affect the *Cordyceps* population.

*Cordyceps,* as virulent to insects’ larvae, maintains the population of moths. Economically, as it is in high demand (as discussed in the review by [14]), gradual improvements in the economic status of people residing at higher elevations have been observed due to the benefits derived from this species. Also having enormous therapeutic potential, this species may help revolutionize the discovery of different pharmacological compounds. Moreover, knowledge on the pathogenicity factors of this fungus may allow us to obtain more insights on the molecular process of infection and may also help us to exploit its behavioral information.

## 10. Concluding Thoughts

The ultimate aim of this review is to shed some insight on the mechanism of infectivity and knowledge on the biological complexity of an important species of the entomopathogens in the area of insect pathology. More molecular-based studies are needed in order to fully understand the mechanisms involved. This focused review on studies elucidating the molecular basis of fungal infectivity offers a better understanding of *Cordyceps* biology which may help us in the exploitation of pharmaceutical bioactive compounds of fungal origin. Evidence for the exact identification of the infection mechanism may help in deciphering the important traits used by this fungus during pathogenicity. Also, the fundamental issue is about unveiling the fungal atrocities against the host immune system. This certainly would provide the much-needed insights on the functional components of the insect-infection mechanism.

## 11. Summary Points

- With tremendous globalization in agriculture, tracing fungal infectivity and elucidating the exact mechanism of fungal dispersal has gained an increased interest.
- Endowed with chemical and pharmacological properties, drugs of high therapeutic importance can be prepared by cultivating *OS*.
- Fungus raised on synthetic substrates (either on silkworm or cereal substrates) may prove to be significantly valuable with reduced dependence on biotic and natural resources, thus reducing the escape pressure.
- Fungal infection of agricultural pests may be a breakthrough achievement and may serve as a promising biological control agent.
- MAPKs play vital roles in regulating fungal development, growth, and pathogenicity.

## 12. Future Issues and Insights

- Anamorphs of *OS* such as *Metarhizium sp.* are widely used as biocontrol agents. The introduction of other anamorphs of *OS* plus other entomopathogenic fungi could also be done to check the spread of agricultural pests. This could prove significantly useful and certainly provides a great revolution in replacing synthetic insecticides.
- Rigorous experimentation on larval cuticle-degrading enzymes plus other enzymes may successfully lead to an improved selection of an *OS* strain that could effectively be integrated for agricultural pest management.
- Transcriptional responses of insect larvae against fungal infection could provide much-needed information on genes induced during pathogen infection in insect larvae.
- Tremendous innovative technologies to hunt for gene-encoding secondary metabolites and chemical entities discovered through activating their silent and orphan gene clusters involved in secondary biosynthesis could also be exploited.
- A search for the gold-standard mycological media to increase this fungus in controlled conditions seems enigmatic, but is highly desired.
- With its enormous biological and clinical attributes, biotechnological inventions of *OS* (natural and lab-cultured strains) may prove very promising and deserve further attention.
